# Refining particle positions using circular symmetry

**DOI:** 10.1371/journal.pone.0175015

**Published:** 2017-04-12

**Authors:** Alvaro Rodriguez, Hanqing Zhang, Krister Wiklund, Tomas Brodin, Jonatan Klaminder, Patrik Andersson, Magnus Andersson

**Affiliations:** 1 Department of Physics, Umeå University, Umeå, Sweden; 2 Department of Ecology and Environmental Science, Umeå University, Umeå, Sweden; 3 Department of Chemistry, Umeå University, Umeå, Sweden; Universidade de Mogi das Cruzes, BRAZIL

## Abstract

Particle and object tracking is gaining attention in industrial applications and is commonly applied in: colloidal, biophysical, ecological, and micro-fluidic research. Reliable tracking information is heavily dependent on the system under study and algorithms that correctly determine particle position between images. However, in a real environmental context with the presence of noise including particular or dissolved matter in water, and low and fluctuating light conditions, many algorithms fail to obtain reliable information. We propose a new algorithm, the Circular Symmetry algorithm (*C-Sym*), for detecting the position of a circular particle with high accuracy and precision in noisy conditions. The algorithm takes advantage of the spatial symmetry of the particle allowing for subpixel accuracy. We compare the proposed algorithm with four different methods using both synthetic and experimental datasets. The results show that *C-Sym* is the most accurate and precise algorithm when tracking micro-particles in all tested conditions and it has the potential for use in applications including tracking biota in their environment.

## Introduction

Tracking micro-particles with computer-enhanced video microscopy is a common technique in biophysics, micro-fluidics and colloidal research [1–3]. By monitoring the movement of a particle using: fluorescence microscopy [4], brightfield microscopy [5] or darkfield microscopy [6]; important information of the system under study can be revealed. Biological systems that have been investigated using these methods are for example; mechanical properties of polymers [4,5], diffusion of individual proteins [7], dynamic properties of DNA and interactions of DNA with different molecules [8,9]. Tracking micro-particles is also common when designing micro-fluidic devices [10].

To obtain reliable data of motion, it is important that particle positions are accurately determined [11]. Accuracy is limited by the detection algorithm [12] and the microscope spatial resolution, which is determined by the quality of the optics and the wavelength of the light. To improve accuracy several algorithms have been developed during the years: e.g., Center-of-Mass (*CoM*) [13], Gaussian fitting (*GFit*) [14,15], Cross-Correlation (*XCorr*) [16,17], quadrant interpolation (*QI*) [18–20] and the Hough transform (*CHT*) [21]. Though these algorithms are commonly used, they all have important limitations.

The *CoM* algorithm locates particle centers by selecting an intensity threshold value and generating a binary mask to evaluate the pixel positions of interest. This algorithm is fast and simple, but very sensitive to shot noise and light fluctuations [22]. The *GFit* method is based on approximating the point spread function (the spatial intensity distribution) of a particle with Gaussian functions. The main limitations of this algorithm are that it is sensitive to defocus and that particles much larger than the wavelength of light cannot accurately be described by a Gaussian distribution. The *XCorr* algorithm is based on locating objects by correlating different radial profiles to find the particle center of symmetry [23]. This algorithm is sensitive to noise and interference patterns from surrounding particles. The *QI* algorithm takes advantage of the circular geometry of non-diffraction-limited objects and uses image interpolation to achieve subpixel accuracy. *QI* requires, however, an accurate initial estimation of the particle position to perform well. Finally, the *CHT* algorithm is commonly used for detecting geometrical patterns and several modifications have been made of the algorithm to make it faster and more robust against noise; e.g., edge-drawing circles [24], isosceles triangle circle detection [25,26], and ellipse detection [27,28]. The main advantage of *CHT* is that it is capable of handling occlusions. However, it is still more sensitive to noise than algorithms not based on edge features, and its accuracy is dependent on the size of the circular pattern.

To improve the accuracy and precision of particle location we present a new approach, the Circular Symmetry (*C-Sym*) algorithm. The algorithm uses correlation analysis to determine the degree of symmetry. We hypothesize that, by using spatial symmetry, the particle position can be more accurately determined even in the presence of significant noise. General symmetry have previously been used and evaluated using techniques such as; phase information [29], symmetry kernels [30], graphs [31], clustering [32], or partial medial axis segments [33]. However, the *C-Sym* technique uses a different scheme: by subsequently employing spatial filtering, piecewise Hermite interpolation and polynomial fitting can we achieve subpixel accuracy and improve the robustness? We evaluated the performance of *C-Sym* using: synthetic images that simulate spherical particles in different conditions; and experimental data of micro-spherical particles in a bright field microscope. The results of *C-Sym* algorithm are compared with that of *CHT*, *CoM*, *XCorr*, *QI* and *GFit* algorithms. The results show that *C-Sym* has better accuracy and precision, especially when tracking particles in environment with noise.

## Materials and methods

### Design of the experiment with synthetic light microscopy images

To evaluate the accuracy and precision, defined as the magnitude of the error and the spread of the error, in particle location we used computer generated (synthetic) images based on the intensity profile of particles under light microscopy. This allowed us to have control over the experimental parameters without the errors introduced by experimental equipment; e.g., imaging system [34,35]. The datasets were, however, not used to build the *C-Sym* algorithm but to validate the performance.

A preliminary analysis indicated that particle size and noise level are variables with a strong impact on particle position estimation. Thus, in our experiments we independently tested both variables by generating particles with a known radius and then exposed the images to white-noise using a zero mean Gaussian distribution. We generated 1000 test simulations for each particle size and noise level, using the signal-to-noise-ratio (SNR) defined as,

$$SNR=\frac{I_{\text{max}}-I_{\text{min}}}{4\sigma }-1,$$ (1)

where *I*_max_ and *I*_min_ are the maximum and minimum intensities of the image, and *σ* is the standard deviation (SD) of the noise signal in the image. Examples for different SNR are shown in S1 Fig, and the Matlab code to generate the synthetic images can be found in the Supporting Information section S1 Dataset.

**Fig 1:**
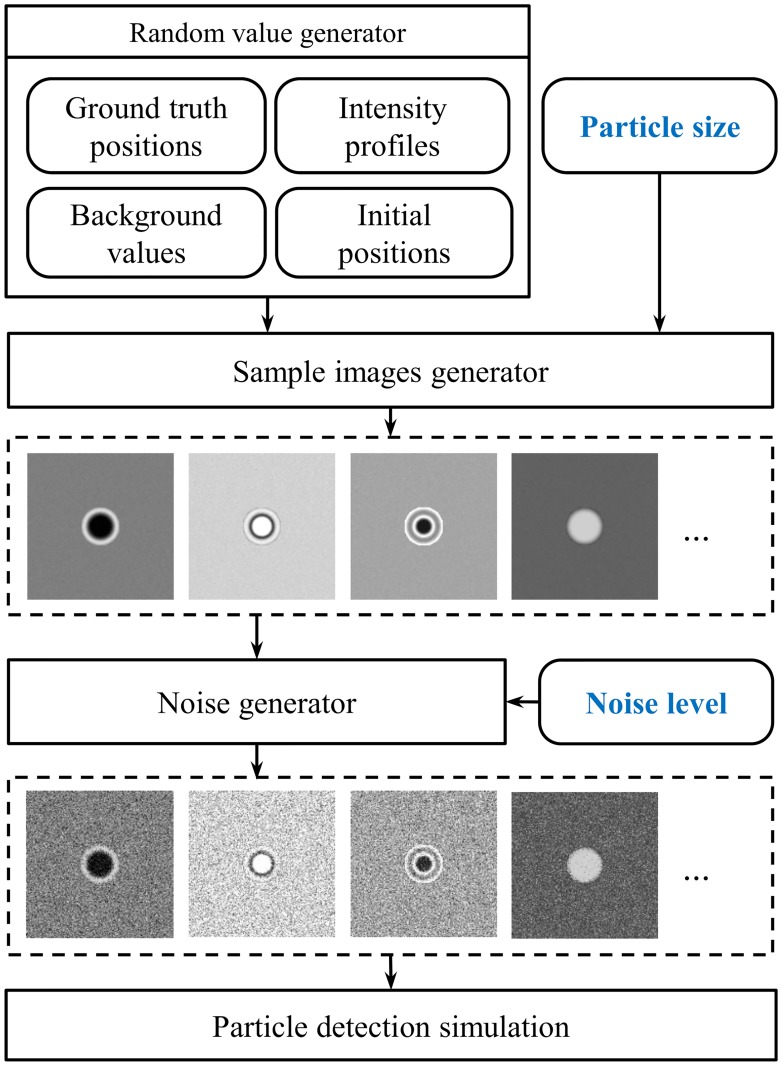
The block diagram of generating sample images. Two major variables, particle size and noise level, and other relevant variables were used to create synthetic images.

Additionally, we identified several other variables that slightly influenced the particle position estimation and could act as a source of bias in the analysis, i.e., particle structure, ground truth position, estimated initial position, and background color. The particle structure, i.e., the visual appearance in the image, can vary considerably according to the experimental setup and the nature of the particle itself. This can significantly change the accuracy of the algorithm used for position estimation. We generated different patterns representing micro-particles, which varied from a simple “spot” to diffraction patterns, see Fig 1. The diffraction patterns were generated using a diffraction profile extracted from a real particle and by introducing random variations such as; inversion of color, profile scaling and stretching. To avoid bias when generating synthetic images, we set the ground truth position as a random floating-point pixel position. For the initial estimation of particle position, we introduced a random error up to 2 pixels to simulate the labeling or segmentation error. Finally, the background intensity of each image was randomly selected from values between 0.25 and 0.75 representing typical test conditions. Randomized synthetic images (512x512 pixels) for each particle size with the above mentioned variables were generated. In addition, we added Gaussian noise with zero means and variance σ^2^ to the images. A schematic diagram showing the generation of test images of certain particle size and noise level is shown in Fig 1. Additional reference particles are shown in the Supporting Information in S2 Fig.

In summary, to avoid biased results we conducted 1000 trials for each particle size and noise level using: randomized background colors, particle patterns and particle positions with a constant region of interest (ROI) size (set to 1.2 times the generated particle size). Each algorithm was evaluated in terms of its accuracy and precision using the mean and standard deviation of the Euclidean distance between the estimated position of the particle and the ground truth.

Besides, we evaluated the accuracy and precision of two overlapping particles by generating synthetic images of two diffraction patterns interfering with each other. This simulation was conducted using two identical particles created by a Bessel function, varying the distance of the particles and the level of noise. The results of this experiment are located in the Supporting Information S1 File.

### Design of the experiment with synthetic fluorescent images

To investigate the influence of patterns similar to fluorescent microscopy, when finding a particle position, we simulated images using an 2D Gaussian distribution function defined as,

$$g\left(x,y\right)=g_{0}\;e^{-\left(\frac{\left(x-\mu_{x}\right)}{2\sigma_{x}{}^{2}}+\frac{\left(y-\mu_{y}\right)}{2\sigma_{y}{}^{2}}\right)},$$ (2)

where g_0_ is the amplitude of the function and represents intensity in the center of the particle, (*μ*_*x*_, *μ*_*y*_) is the mean of *x* and *y*, and represent the center of the particle, and (*σ*_*x*_, *σ*_*y*_) is the standard deviation of *x* and *y* and represent the size of the particle.

We conducted the experiments changing the particle size and the image noise, using a constant ROI with a size of 1.2 times the standard deviation of the generated particle, following the procedures in the previous section.

### Design of the experiment with micron-sized particles

**Fig 2:**
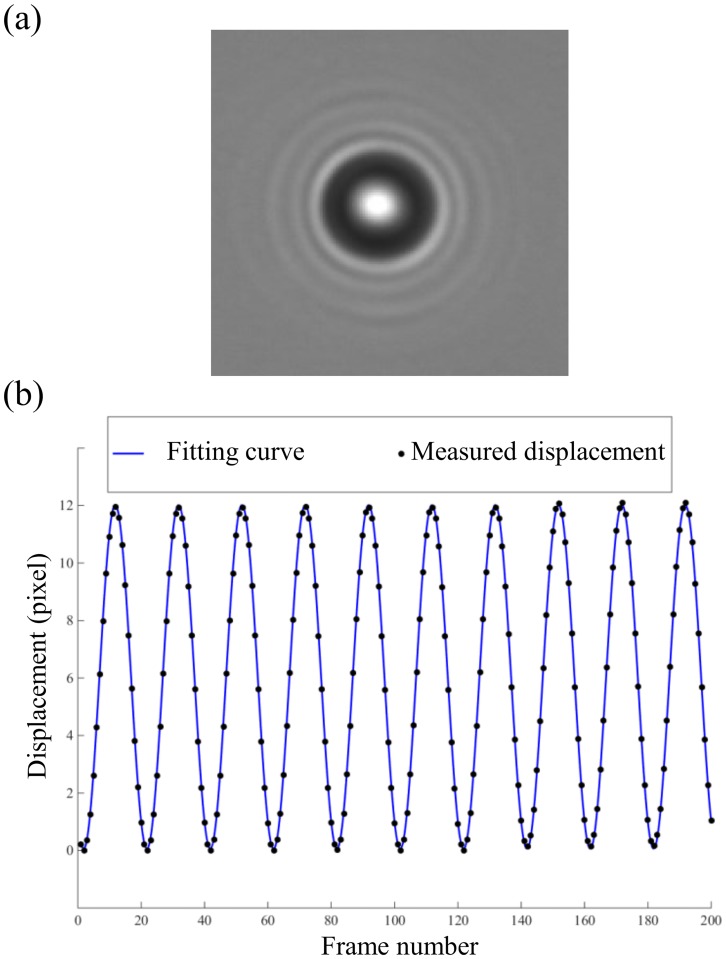
(a) Example of an image from the experiment conducted with a 2.0 μm silica micro-sphere immobilized to a cover slip. (b) Measured displacement of the particle with the proposed *C-Sym* algorithm (marked as circles) and the ground truth sinusoidal function for the first 200 frames of the experiment (solid line).

To validate *C-Sym* we also generated experimental data by oscillating a micron-sized particle in a bright field microscope and evaluated the error of amplitude (peak-to-peak displacement distance) by comparing the tracked position with the real piezo-stage motion. We prepared our sample using silica micro-spheres (Catalog Code SS04N, Manufacturer Lot Number: 7829 –Bang Laboratories) with a diameter of 2.0 μm suspended in a solution of Milli-Q water. Micro-spheres were immobilized to a cover slide by drying 10 μl of the suspension in room temperature. The samples were then placed in a microscope (Olympus, IX71), modified for optical tweezers and flow chamber experiments, and imaged using a water immersion objective (UPlanSApo 60x, Olympus) [36,37]. A representative image of a micro-sphere is shown in Fig 2a. The micro-spheres were moved by a piezo stage with sub-nm resolution (PI-P5613CD, Physik Instruments). We used a CCD camera (C11440-10C, HAMAMATSU, 8 bit) with a conversion factor (1 px = 84.7 ± 0.5 nm, mean ± standard error (SE) on the mean, and the standard deviation (SD) was 1.6 nm), to record the motion.

To obtain interpretable results, we compared the displacement of the piezo stage with the displacement obtained by fitting detected particle positions to a sinusoidal function

f(i)=asin(2πi/b+2π/c)+d, (3)

where *i* is the frame number, *a* is the amplitude of displacement in pixels, *b* is the period, *c* represents the phase and *d* is an offset. In this case, *b* is a constant defined by multiplying the cameras sampling speed and the piezo stage oscillation period.

To transform the displacement in pixels to real space distance, an additional calibration process is required. In an ideal projection system, like a pin-hole camera, distances from the image plane can be transformed to the real plane of interest using a multiplication factor. However, when using an imaging system a more complex calibration model is required to deal with plane misalignment [38] or lens aberrations [39], and other systematic errors of the instruments [40], For simplicity, we modelled the image to real-space-projection as a parametric polynomial function. Based on our results, we choose a 5^th^ degree parametric polynomial function depending on the amplitude of the displacement expressed as,

$$D=p_{6}a^{5}+p_{5}a^{4}+p_{4}a^{3}+p_{3}a^{2}+p_{2}a+p_{1},$$ (4)

where *D* is the real amplitude of the particle displacement in nm, and *p*_i_ are the calibration parameters. We used 11 video sequences with a known *D*. Thereafter, all algorithms were used to measure the amplitude *a* from the videos, and the calibration parameters *p*_i_ were calculated by minimizing the projection error (the distance between the projection of measured amplitude *a* and *D*). By applying this approach, the resulting projection error was <1% and the correlation with the ground truth was statistically significant (t-test, p<0.05) and with a correlation value >0.999.

### Design of the experiment with tethered particles

**Fig 3:**
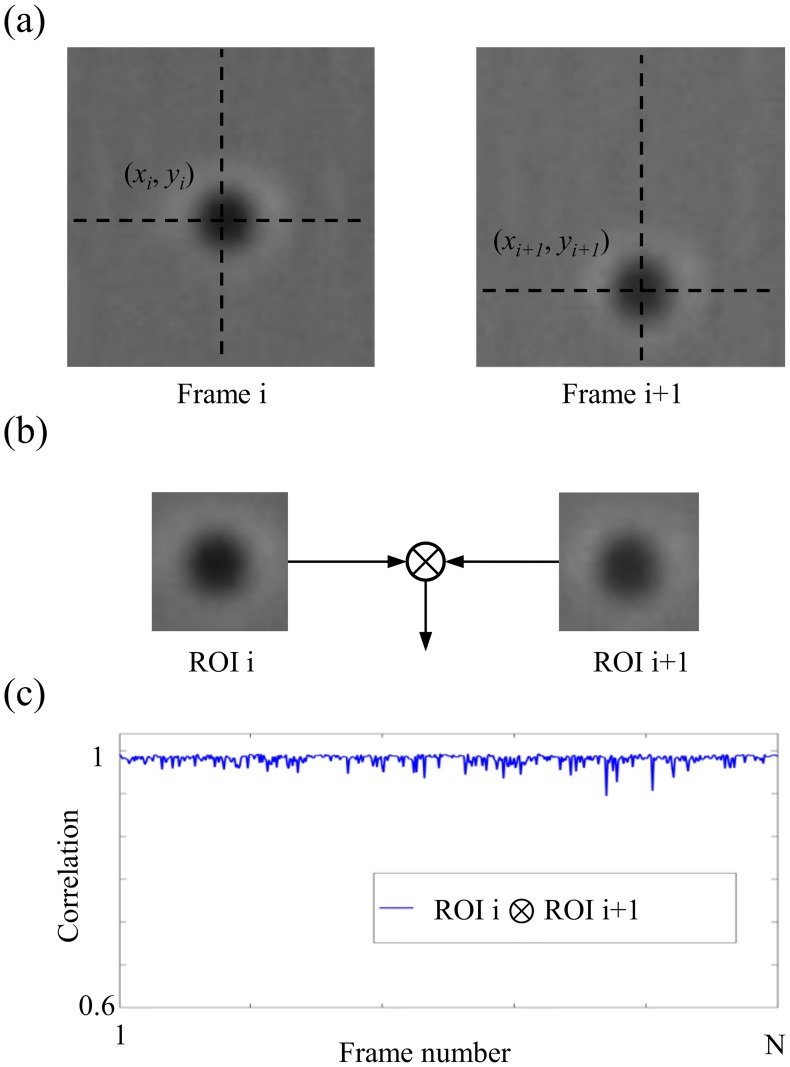
Evaluation of the performance of the algorithms using a tethered micro-sphere. (a) The algorithms are used to locate the micro-sphere position in each frame. (b) The micro-sphere is extracted from every frame using a constant sized ROI centered on the detected position. Consecutive ROIs are correlated as denoted by the operator ⊗. (c) The correlation for each frame number. An algorithm with poor precision will give a low correlation value, thus, the evolution of correlation is an indicator of the stability and robustness of the algorithm used to locate the micro-sphere.

Using bright field experimental data of supercoiled tether DNA, we tested the accuracy of *C-Sym* in comparison to the other algorithms. The video data was taken from the studies in reference [41], In these experiments, supercoiled DNA (pSB4312) was immobilized at one end to a coverslip using PNAs while the other end was attached to a 0.51 μm Streptavidin-coated micro-sphere (cat. no. CP01N, Bangs Laboratory). The motion of a micro-sphere was recorded at 225 Hz in an inverted microscope (Model No. DM IRB, Leica) with a high-speed camera (Pike F100B, Allied Vision Technology, conversion factor of 49.97 nm per pixel). The length of the time series were optimized to 30 seconds according to an Allan variance analysis of the setup [42], Representative images (62x62 pixel ROI) are shown in Fig 3a.

In these experiments, no ground truth of the particle position was available since a micro-sphere will move stochastically due to the Brownian motion. However, since the appearance of the microsphere in each frame, Fig 3a, only marginally changed from frame to frame because the motion is more or less constrained in a plane, the algorithms were applied to each frame to extract particle positions. A ROI of fixed size was applied around the detected particle in each frame, see Fig 3b. The ROI from frame *i* was thereafter correlated with ROI_*i*+1_ resulting in a number between 0 and 1, where the latter corresponds to perfect correlation. The consecutive correlation time series of an experiment is shown in Fig 3c. Thus, if an algorithm is able to accurately predict the position of the particle, the correlation value should be close to 1. Note that this does not provide a direct measurement of accuracy for the algorithms; however, it measures the similarity of ROIs with the particle positioned in the center and therefore reveals the accuracy and precision of particle location.

In the original video sequences, we estimated the mean SNR of frames to be 10. To test the algorithms for robustness against noise, we applied to each frame white-noise with zero mean and different variance, σ, to obtain the mean SNRs in the range from 0.1 to 10. At each noise level we evaluated each algorithm using the relative difference of correlation values. The particle position in a noisy image were found by the algorithms and used in the original image as (*x*_*i*_.*y*_*i*_), see procedure in Fig 3.

## Central-Symmetry algorithm (*C-Sym*)

**Fig 4:**
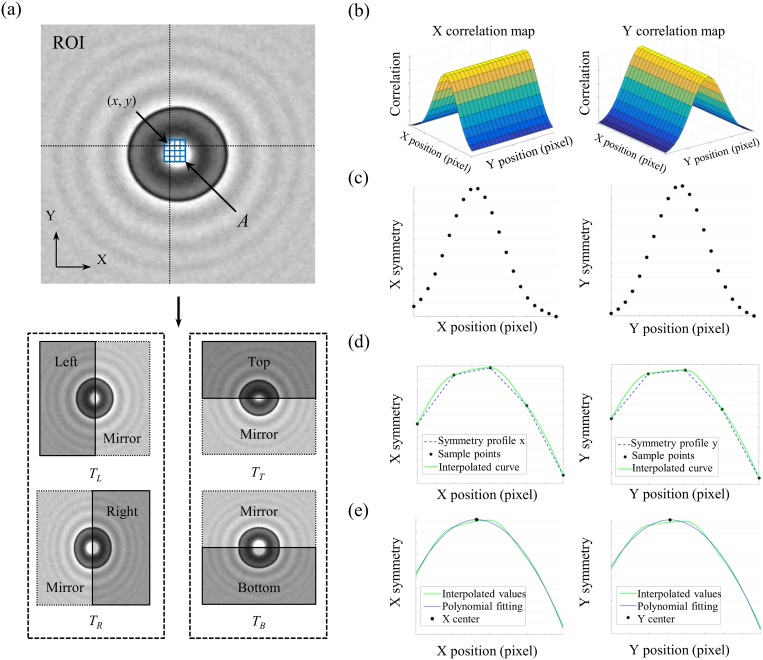
The workflow of the *C-Sym* algorithm. (a) For candidate points (*x*, *y*) in a search area A, a region of interest (ROI) is defined and four templates of the particle are created, dividing the ROI horizontally and vertically and reconstructing the whole particle from each template. (b) Pairs of templates are used in Eqs 5 and 6, to create the 3-D correlation maps, *Corr*_*X*_ and *Corr*_*Y*_. (c) 2-D symmetry profiles, *Sym*_*X*_ and *Sym*_*Y*_, are created from correlation maps using average filtering defined by Eqs 7 and 8. (d) Symmetry profiles are interpolated using the Hermite algorithm. (e) Correlation centers are obtained from interpolated Symmetry profiles with polynomial fitting.

Accurately extracting the particle position from images can be problematic if the images have: a substantial amount of noise, changing background colors, or changing particle appearance. Our approach to handle this, is to use geometrical symmetry to find the particle position. A rough estimate of the particle position is first needed. This can be obtained using standard methods, e.g., manual selection, template matching, or segmentation, but the choice of method is not crucial for the performance and the final results. Since the algorithm consist of several steps, we present these steps in the workflow chart in Fig 4, and each step is described below.

**(a) Template extraction:** a search area *A*, defined as a two dimensional array, is positioned around the initial center estimation, see Fig 4a. For each point (*x*, *y*) in *A*, a ROI is created with a fixed size (*n×n)* defined by the user. In general, this ROI should be slightly larger than the particle. The ROI is divided vertically at *x*, and a mirror image of each half is; created, flipped and concatenated to each half to form two templates denoted *T*_*L*_ and *T*_*R*_. A similar process is conducted horizontally at *y* resulting in two new templates, *T*_*T*_ and *T*_*B*_, as shown in Fig 4a. The templates denoted *T*, are all functions of *x* and *y*, (i.e., $T_{L}^{X,Y}$), for clarity we choose not to write that dependency explicitly.
**(b) Symmetry measurement:** the 3D correlation map, *Corr*_*X*_(*x*, *y*), is derived from the template pair *T*_*L*_ and *T*_*R*_, representing the vertical symmetry of the particle at the candidate point *(x*, *y)* in *A*. Likewise, the 3D correlation map *Corr*_*Y*_(*x*, *y*) is derived from *T*_*T*_ and *T*_*B*_, representing the horizontal symmetry of the particle at (*x*, *y*). The correlation maps are defined as,
  $$Corr_{X}\left(x,y\right)=\frac{\sum_{i,j}^{nxn}\left(\left(T_{L}^{}\left(i,\text{j}\right)-\mu_{L}^{}\right)\left(T_{R}^{}\left(\text{i},\text{j}\right)-\mu_{R}^{}\right)\right)}{\sigma_{L}^{}\sigma_{R}^{}},$$ (5)
  $$Corr_{Y}\left(x,y\right)=\frac{\sum_{i,j}^{nxn}\left(\left(T_{T}^{}\left(i,\text{j}\right)-\mu_{T}^{}\right)\left(T_{B}^{}\left(\text{i},\text{j}\right)-\mu_{B}^{}\right)\right)}{\sigma_{T}^{}\sigma_{B}^{}},$$ (6)
  where *T*_*L*_, *T*_*R*_, *T*_*T*_, *T*_*B*_ represent the four templates of size *n×n* built around the coordinates (*x*, *y*); and *μ* and *σ* are the average and standard deviation of the corresponding template.
**(c) Dimensional filtering:** To increase the robustness against noise an average filter is applied in each 3D correlation map. This reduces the correlation maps into two 2D symmetry profiles, *Sym*_*X*_ and *Sym*_*Y*_, as shown in Fig 4c and defined by
  $$Sym_{X}\left(x\right)=\frac{1}{N}\sum_{y=1}^{N}Corr_{X}\left(x,y\right),$$ (7)
  $$Sym_{Y}\left(y\right)=\frac{1}{N}\sum_{x=1}^{N}Corr_{Y}\left(x,y\right)\;,$$ (8)
  *Sym*_*X*_ and *Sym*_*Y*_ will have a maximum when *T*_*L*_ and *T*_*R*_, and *T*_*T*_ and *T*_*B*_ are identical. This maximum represents the center of symmetry of the particle, thus providing the center position.
**(d) Piecewise Hermite interpolation:** To improve the accuracy, we use the Hermite piecewise algorithm [43], which allows us to increase the resolution of the symmetry profiles and to find the maximum in *Sym*_*X*_ and *Sym*_*Y*_ with subpixel accuracy [44]. The advantage of using piecewise Hermite interpolation in our algorithm is shown and discussed in the Supporting Information S1 File.
  In the interpolation process for *Sym*_*X*_, the discrete points *x*_1_, *x*_2_, …, *x*_*n*_ are used to create a set of third-degree polynomials defined as,
  $$p_{k}^{x}\left(t\right)=\left(2t^{3}-3t^{2}+1\right)x_{k}+\left(t^{3}-2t^{2}+t\right)m_{k}+\left(-2t^{3}+3t^{2}\right)x_{k+1}+\left(t^{3}-t^{2}\right)m_{k+1},$$ (9)
  where *t* are values in the interval [*x*_*k*_, *x*_*k+*1_]*; m*_*k*_ is the slope of the tangent line of *Sym*_*X*_ at *x*_*k*_ and *m*_*k+*1_ is the slope of the tangent line of *Sym*_*X*_ at *x*_*k+*1_ respectively. The same procedure is applied to the Y axis. The results of this process are two continuous curves with a continuous first derivative which passes through the sampled values of *Sym*_*X*_ and *Sym*_*Y*_ respectively. These continuous curves are denoted as *S*_*X*_ and *S*_*Y*_.
**(e) Peak analysis:** The continuous curves *S*_*X*_ and *S*_*Y*_ represent the symmetry of the particle in *A* and their peaks represent the X and Y coordinates of the particle respectively. To find these peaks, 2^nd^ degree polynomials are fitted to *S*_*X*_ and *S*_*Y*_ using 500 samples located in the neighborhood of the discrete peak and with a sample interval of 0.01 pixels. Each polynomial equation was solved using a Vandermonde matrix. Once the polynomial is obtained, the center is calculated using the equation defined as,
  $$pol\left(x\right)=q_{1}x^{2}+q_{2}x+q_{3},\;$$ (10)
  where *q*_*i*_ are the polynomial parameters. The center of the particle in the X-axis, *c*_*X*_ *= −q*_2_ / 2*q*_1_, is the peak of the fitted polynomial. The same procedure is used to locate the center of the particle in the Y-axis. When the Hermite interpolation is omitted, the 2^nd^ degree polynomials are fitted to *Sym*_*X*_ and *Sym*_*Y*_ using 5 discrete samples in the neighborhood of the peak.

## Results and discussion

The performance of the *C-Sym* algorithm in the particle location experiments with simulated and real particles are outlined in detail below. These synthetic and experimental datasets were however not used to build our *C-Sym* algorithm but instead used to compare *C-Sym* with five commonly used algorithms: *CHT*, *CoM*, *XCorr*, *QI* and *GFit*, details of these latter algorithms are described in the Supporting Information S1 File.

### Experiment with synthetic light microscopy images

**Fig 5:**
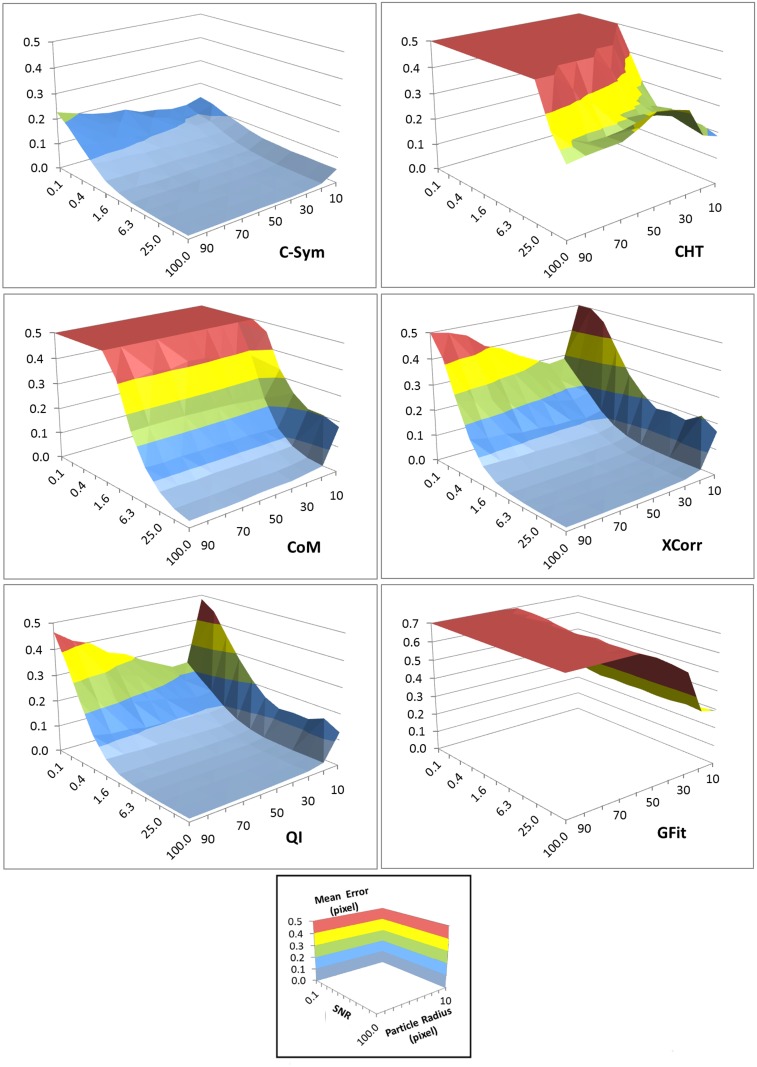
Mean error in the center position of the particles measured using: *C-Sym*, *CoM*, *CHT*, *XCorr QI* and *GFit* algorithms for different particle radius and noise levels. The bottom panel shows the scales and label of each axis. A low value of the SNR indicates noisy images. S1 and S2 Figs show examples of the images used to generate these data.

**Fig 6:**
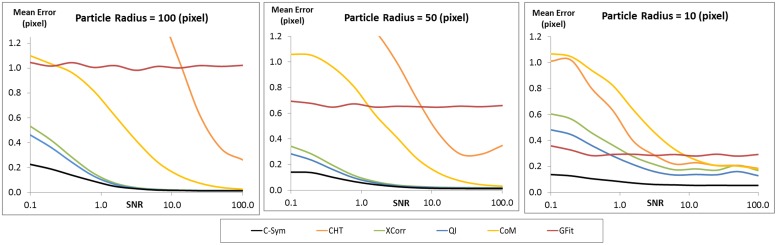
Comparison of the mean error in the center position of a particle of constant radius for different SNR values. S1 Fig 1–2 show examples of the images used to generate these data.

In line with our hypothesis, *C-Sym* showed better accuracy and precision, especially for noisy images. The evaluation was performed by using 110 000 simulated particle images with a resolution of 512x512 pixels and by varying the SNR from 0.1 to 100 in 11 steps. The particle radii varied from 10 to 100 pixels in 10 steps in these images. Estimated errors for each fixed particle size and SNR are shown in Fig 5. To further illustrate the effect of SNR and particle radius, we chose three selections of the results with a constant particle radius at 10, 50 and 100 pixels as shown in Fig 6.

Both *CHT* and *CoM* showed lower accuracy than the other algorithms for SNR lower than 50, i.e. for images with more noise. *GFit* showed low accuracy for diffraction patterns in large templates, that is >30. However, *XCorr*, *QI*, and *C-Sym*, all performed well down to a SNR of 1. Here, *QI* was slightly better than *XCorr*, whereas *C-Sym* was better than both the others and able to provide high accuracy, 0.2 pixels mean error, at a SNR of only 0.1.

Particle size had a more complex effect on the result; implying for example that bigger particles are more influenced by noise. The response of *CHT* and *GFit* showed high error values for some particle sizes even in low noise levels and *CoM*, *XCorr* and *QI* generated low accuracies for particles smaller than 20 pixels in radius. However, *C-Sym*, does not show this limitation, achieving a maximum mean error close to 0.1 pixel with particles of 10 pixel radius.

**Fig 7:**
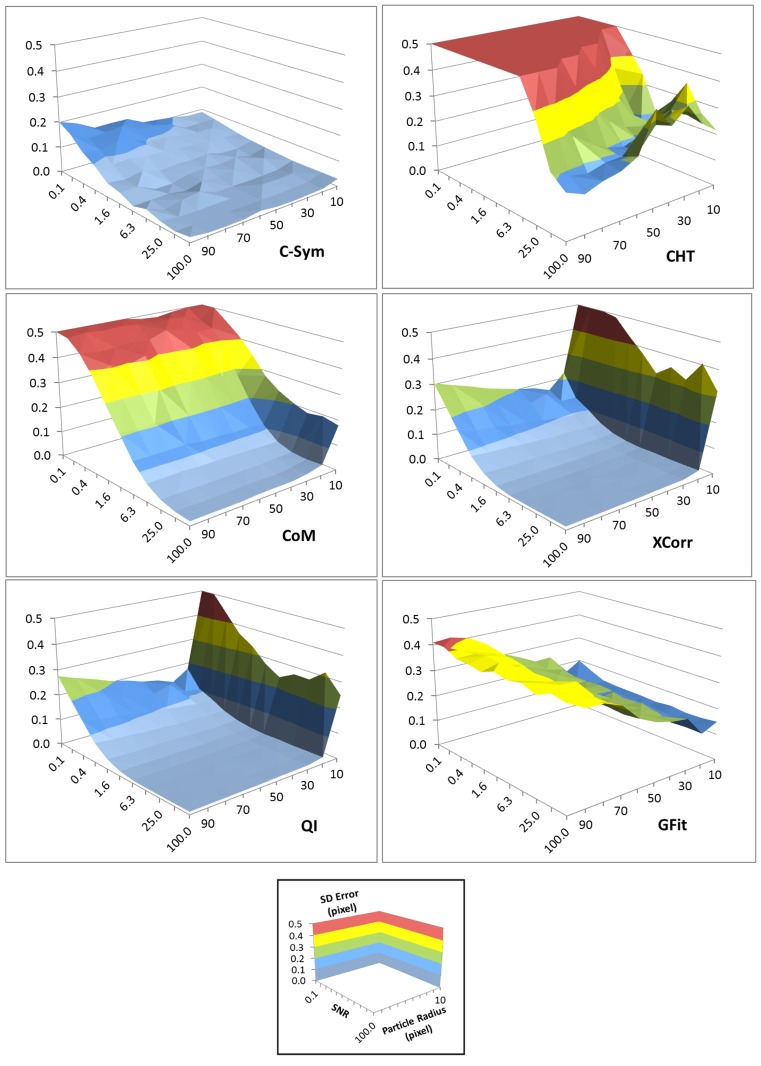
Standard deviation of the error in the position of the center of particles measured with *C-Sym*, *CHT*, *CoM*, *XCorr*, *QI* and *GFit*, algorithms according to the particle radius and the noise level. S1 and S2 Figs show examples of the images used to generate these data.

**Fig 8:**
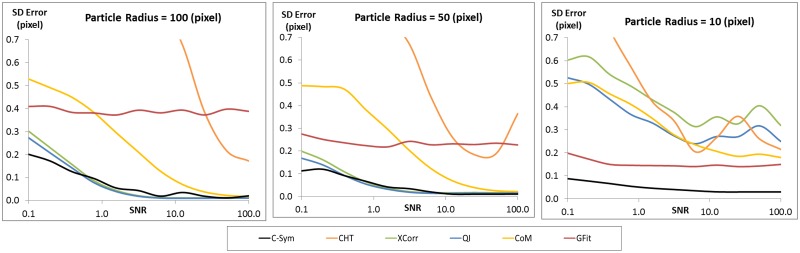
Comparison of how the standard deviation of the error in the position of the center of particles measured with different algorithms change with noise level while using a constant particle radius of 100 (left) 50 (center) and 10 (right) pixels. S1 and S2 Figs show examples of the images used to generate these data.

The precision of each algorithm was evaluated using the Standard Deviation (SD) of error in particle position (Fig 7) and Fig 8 shows three selections of the results with a constant particle radius at 10, 50 and 100 pixels. The precision results were very similar to the accuracy results. In particular, precision of *CHT* and *CoM* decreased significantly with noise, and the precision of *GFit* decreased significantly with particle size, whereas *XCorr* and *QI* showed the best precision with large particles and low noise levels. For these conditions, *C-Sym* showed similar precision to *QI*, however, *C-Sym* outperformed *QI* for small particles and high noise levels.

In conclusion, we have shown that *CHT* and *GFit* is, in general, inaccurate when measuring particle positions. *CoM* has the disadvantage of being very sensitive to noise and *GFit* is sensitive to the size of diffraction pattern. Yet, *XCorr* and *QI* perform better, where *QI* slightly outperformed *XCorr*. This finding was expected, because *QI* uses *XCorr* in its first step and thereafter refines the results. These results are also consistent with previous findings [45], However, our data suggest that *QI* is not significantly better than *XCorr*. Overall, *C-Sym* showed the best results, achieving generally similar or better accuracy and precision than the compared algorithms, while still being able to measure smaller particles and being more robust against noise.

### Experiment with synthetic fluorescent images

**Fig 9:**
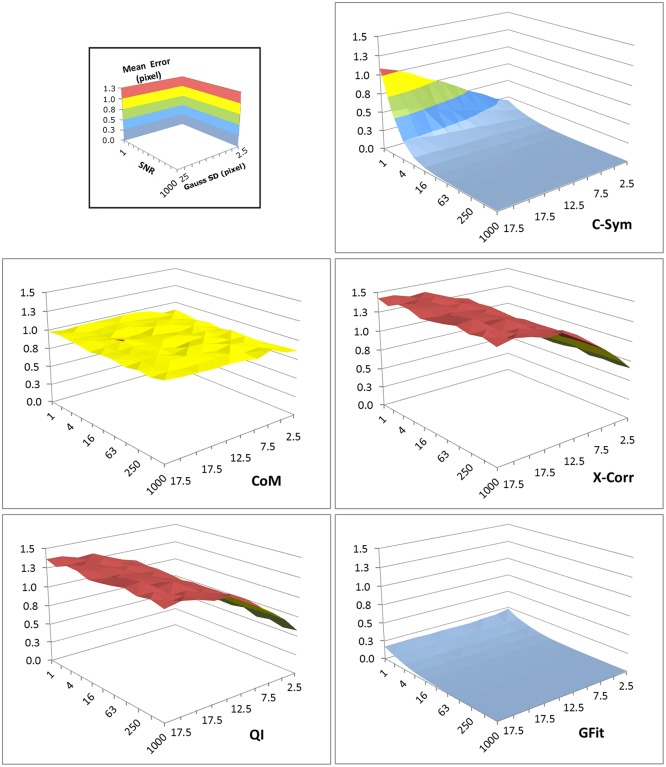
Mean error in the center position of the particles measured using: *C-Sym*, *CoM*, *XCorr QI* and *GFit* algorithms for synthetic fluorescent images using different particle radius (presented in terms of the standard deviation of Gaussian distribution) and noise levels. S3 Fig show examples of the images used to generate these data.

**Fig 10:**
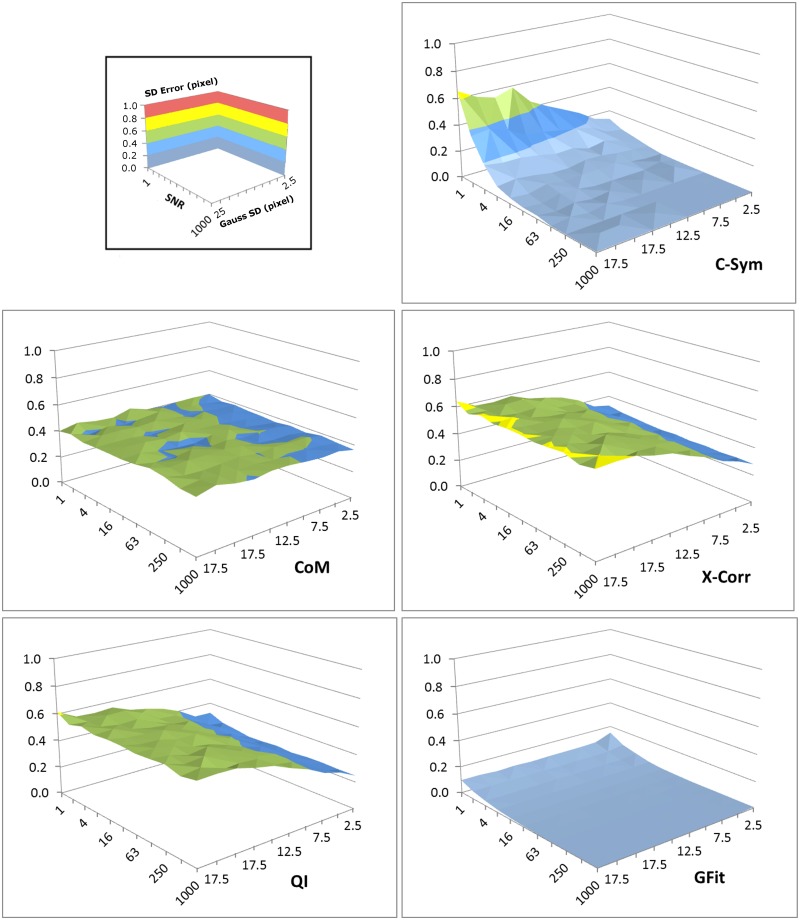
Standard deviation of the error in the position of the center of particles with *C-Sym*, *CoM*, *XCorr*, *QI* and *GFit*, algorithms according to the particle radius (presented in terms of the standard deviation of Gaussian distribution) and the noise level. S3 Fig show examples of the images used to generate these data.

**Fig 11:**
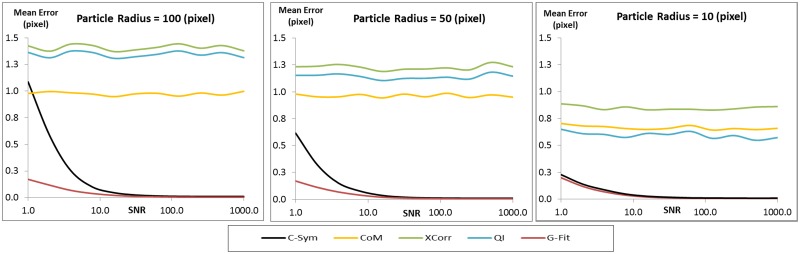
Comparison of how the mean error in the position of the particle centers measured with different algorithms change with noise level while using a constant particle radius of 100 (left) 50 (center) and 10 (right) pixels. S1 Fig 3 show examples of the images used to generate these data.

**Fig 12:**
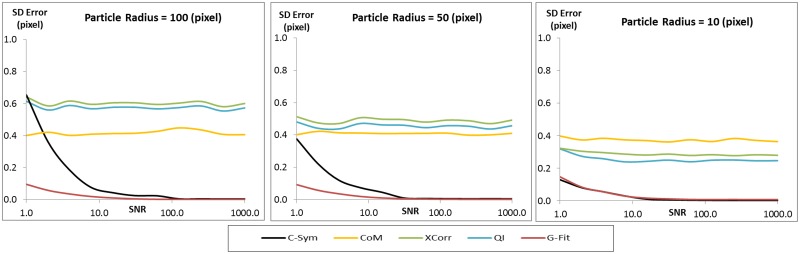
Comparison of how the standard deviation of the error in the center position of particles measured with different algorithms change with noise level while using a constant particle radius of 100 (left) 50 (center) and 10 (right) pixels. S1 Fig 3 show examples of the images used to generate these data.

In this experiment, *C-Sym* showed high accuracy and precision at low noise levels. We used synthetic fluorescent like images as shown by a representative data set in S3 Fig. Estimated mean and SD error in particle position are shown in Figs 9 and 10, respectively, and three selections of mean and SD of error with a constant particle radius at 10, 50 and 100 pixels are presented in Figs 11 and 12. *CoM* did not perform well providing an almost flat error surface. This reflects the inability of the algorithm to refine the initial center estimation (containing a ±2 pixels random error). We found that only when using a significantly bigger ROI, where all nonblack pixels of the particle are visible, and a pure black background and moderate levels of noise, the *CoM* is able to obtain a behavior comparable to the other techniques.

*Xcorr* and *QI* showed large errors with increasing particle size. *GFit* achieved the best overall results, which was expected since the particles were generated with the exact model *GFit* uses for fitting. However, *C-Sym* showed relatively low errors in general and achieved almost the same accuracy as the *GFit* at SNRs above 4 regardless of the size of the pattern.

### Experiment with micron-sized particles

**Fig 13:**
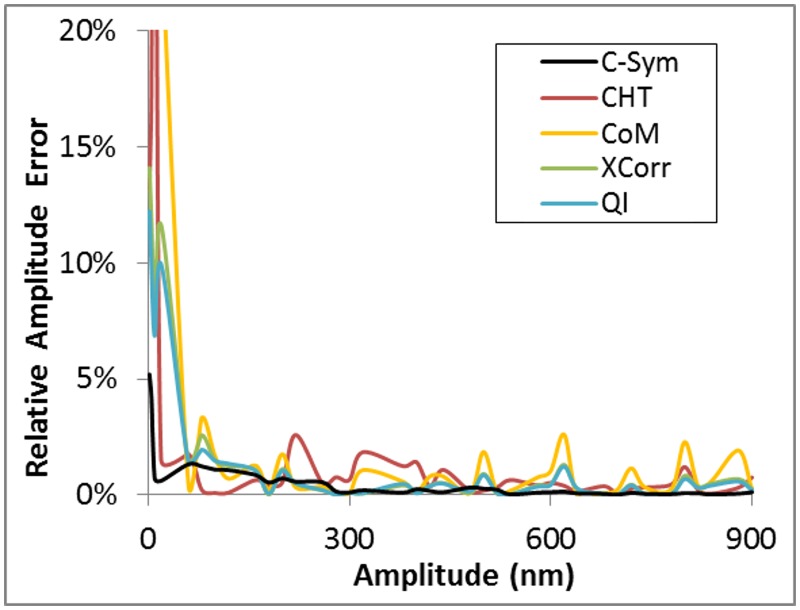
Relative error of the amplitude of particle displacement with *C-Sym*, *CHT*, *CoM*, *XCorr* and *QI*, algorithms.

To validate and compare the performance of *C-Sym* using real data, 40 video sequences with 16 000 frames acquired at 500 Hz of 2 μm micro-spheres attached to a glass surface were analyzed. A micro-sphere was oscillated at 1 Hz with a sinusoidal function and varying peak-to-peak amplitude, from 1 to 900 nm. The estimated SNR of the video sequences is 50. The comparison of the aforementioned algorithms was done by using a relative amplitude error analysis (the absolute error divided by the amplitude of the displacement), as shown in Fig 13 and Table 1. We excluded *GFit* from the result since the error was one magnitude higher than other methods. This is consistent with the results we derived using synthetic transmitted light microscopy images.

**Table 1:** Absolute amplitude errors for the different algorithms in nm.

Algorithm	Absolute Amplitude Error (nm)		
	*Mean*	*SD*	*Maximum*
*C-Sym*	0.71	0.45	1.57
*CHT*	2.43	2.19	9.66
*CoM*	3.55	4.57	18.30
*XCorr*	1.82	1.92	8.11
*QI*	1.67	1.69	7.59

The obtained results were generally consistent with previous finding [13,16,18], *CoM* obtained the worst results, with an mean error >3 nm, and with a relative error >20% for smallest displacements. *XCorr* and *QI* performed better, but they still obtained an error >10% for the smallest displacements. The proposed *C-Sym* algorithm showed significantly better results, achieving an mean and standard deviation of error <1 nm, with a maximum relative error of 5%. Notably, these results do not support the statements in reference [45] which claims that *CoM* was unable to track particles in a similar scenario and that *XCorr* obtains large errors compared to *QI*.

The results are consistent with the synthetic experiments, and indicate that *C-Sym* is more stable and obtains smaller errors than the other algorithms. In this experiment *C-Sym* was the only algorithm able to measure amplitudes of nm order.

### Experiment with tethered particles

**Fig 14:**
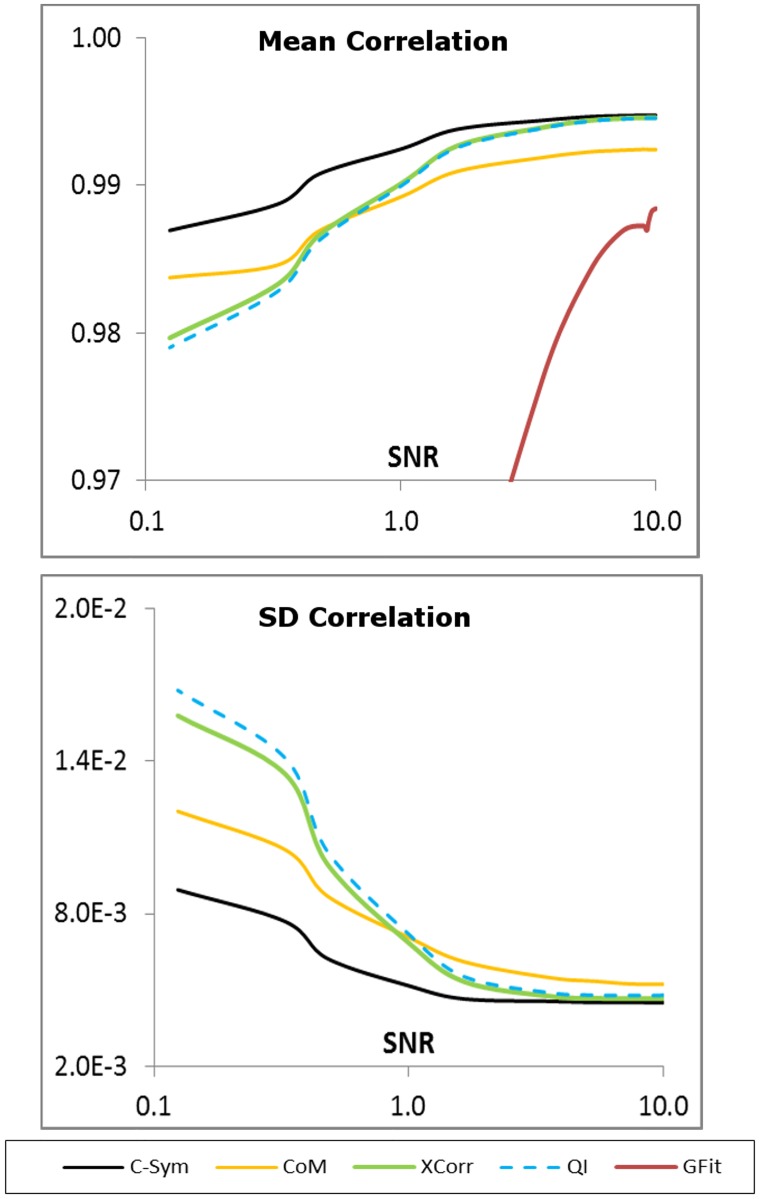
Correlation results in the experiment with tethered particles using *C-Sym*, *CoM*, *XCorr*, *QI* and *GFit* algorithms. For readability, the SD correlation for the *GFit* (two orders of magnitude higher than the other techniques) is not represented in the chart.

Experiments using brightfield video sequences of a micro-particle attached to a coverslip through DNA strands were also conducted. In general, *CHT* and was not able to locate the particle positions, therefore these algorithms were excluded from the comparison. This was expected since the *CHT* requires a well-defined circular pattern; and this is not found in the images since the micro-particles show a diffuse intensity distribution. *GFit* was only able to locate the particle position with accuracy in low noise conditions, and the achieved SD correlation was two orders of magnitude higher than the other techniques. All other algorithms performed well with a correlation >0.95 and a SD correlation <0.02 in all the SNR range as shown in Fig 14. All algorithms also achieve an overall mean correlation >0.99 and SD correlation <0.01, as summarized in Table 2. As in our previous results, *C-Sym* performed overall better than the other algorithms, especially under high noise levels. Noticeably, *QI* showed slightly worse results than *XCorr*, and *CoM* performed better under high noise levels (SNR < 1) than both *XCorr* and *QI*.

**Table 2:** Correlation values for the different algorithms.

Algorithm	Correlation		
	*Mean*	*SD*	*Minimum*
*C-Sym*	0.9932	0.0053	0.8950
*CoM*	0.9905	0.0066	0.8706
*XCorr*	0.9916	0.0067	0.8888
*QI*	0.9915	0.0071	0.8742
*GFit*	0.9577	0.1401	0.0002

## Conclusion

We propose a new algorithm, denoted the Circular Symmetry algorithm (*C-Sym)*, for accurately locating the center of a circular particle. *C-Sym* uses the symmetry of the particle to achieve robust sub-pixel accuracy, capable of handling general circular patterns obtained from images. The strength of the algorithm is that even in noisy conditions, useful information of the particle is kept in the spatial distribution of the symmetry feature.

We compared the algorithm with other state-of-the-art methods: Circular Hough Transform *(CHT)*, Center-of-Mass *(CoM)*, Cross-Correlation *(XCorr)*, Quadrant Interpolation *(QI)* and Gaussian Fitting (*GFit*) algorithms using synthetic and experimental images. The results show that *C-Sym* is more robust and achieves a higher accuracy and precision when measuring particle positions for a wide range of noise levels.

The robustness against noise in images is in particularly useful when studying systems with low light conditions. For example, fast processes that require short shutter times, and optical systems with high f-numbers. In addition, studying fast moving particles in micro-fluidic environments are often subjected to low SNR and particle sizes may vary due to displacements in depth. Therefore, *C-Sym* is expected to be a new useful algorithm for colloidal research, various biophysical systems, giving a reliable estimation of particle location, even though the environment is noisy or the resolution of particles is low.

In this work we have shown that by using 2D spatial symmetry features of micro-particles we can accurately determine their center position. A future work is to expand this symmetry algorithm and investigate if it can find the bilateral axis of symmetry, i.e., the center line of a geometrical object with identical left and right sides. This can prove very useful in several biological applications, e.g., when classifying animals, handling occlusions, and when determining the direction of movement of fish or insects in biological assays.

## Supporting information

S1 Fig Synthetic transmitted light microscopy images at different SNR levels. (TIF)

S2 Fig An example of 30 synthetic light microscopy images. (TIF)

S3 Fig Synthetic fluorescent images with three different sizes (top, middle, bottom) at different SNR levels. (TIF)

S4 Fig Mean error in the center position of the particles measured using *C-Sym*: Four different versions of the algorithm were used to study the influence in the results of a median filtering and of the Hermite interpolation step. (TIF)

S5 Fig Standard Deviation of the error in the position of the center of the particle measured with *C-Sym*: Four versions of the algorithm were used to analyze how a median filtering and the Hermite interpolation step influence the results. (TIF)

S6 Fig Synthetic overlapping images with particle pairs of two different sizes (top and bottom) at different distances from each other ranging from 1 to 3 times the Rayleigh limit *L*. (TIF)

S7 Fig Mean error in the center position of the particles measured using *C-Sym*, *CoM*, *XCorr*, *QI* and *GFit* algorithms according to the particle distances (presented in terms of the multiple of Rayleigh limit *L*) and the noise level. (TIF)

S8 Fig Standard deviation of the error in the center position of the particles measured using *C-Sym*, *CoM*, *XCorr*, *QI* and *GFit* algorithms according to the particle distances (presented in terms of the multiple of Rayleigh limit *L*) and the noise level. (TIF)

S1 File This is the main supporting information document. It contains additional details of the experiment design, the algorithms of this paper, and the additional conducted experiments.
(DOCX)

S1 Dataset Matlab scripts used to generate the synthetic images in the conducted experiments. (ZIP)
