# Supplementary figures and images for: Refining particle positions using circular symmetry
Source: PLoS One. 2017 Apr 12;12(4):e0175015. doi: 10.1371/journal.pone.0175015 (PMC5389671; doi:10.1371/journal.pone.0175015)

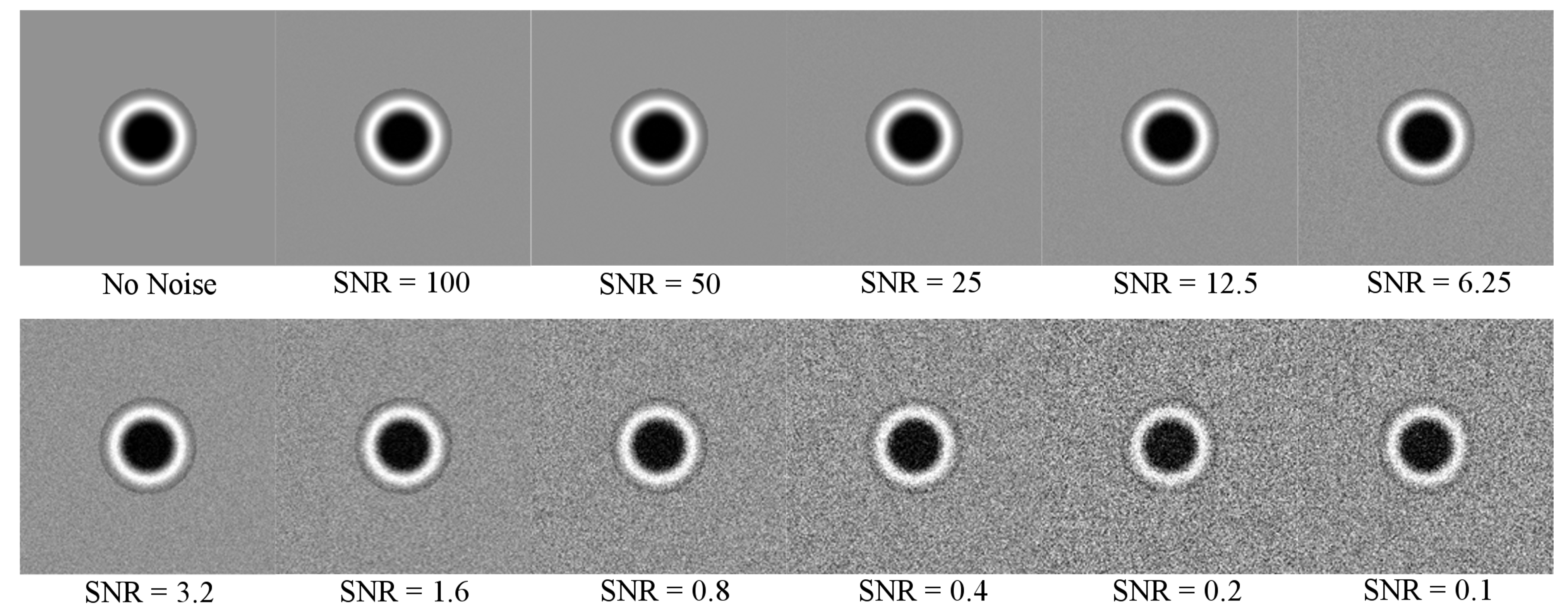

Supplement: S1 Fig — (TIF) [file pone.0175015.s001.tif]

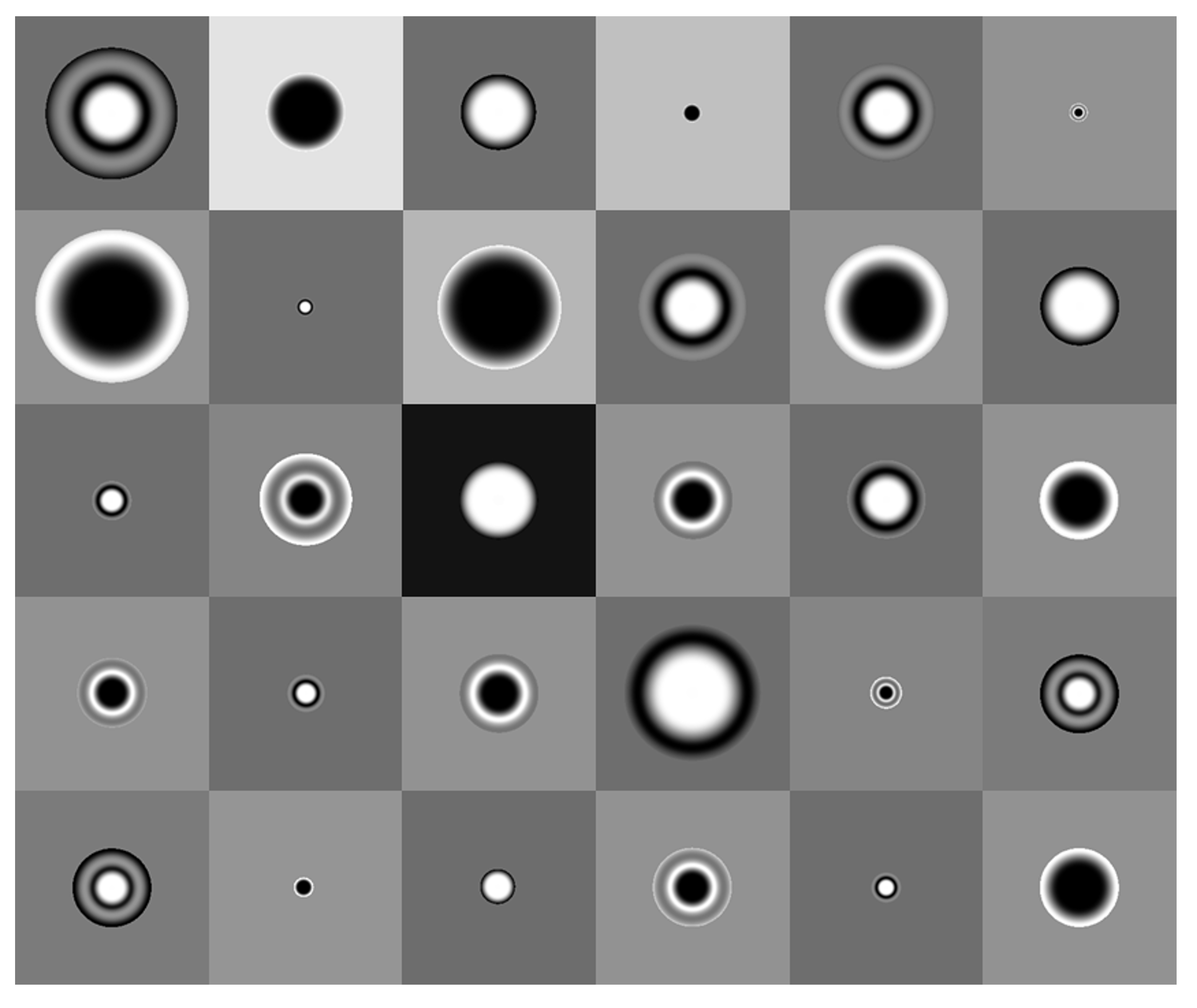

Supplement: S2 Fig — (TIF) [file pone.0175015.s002.tif]

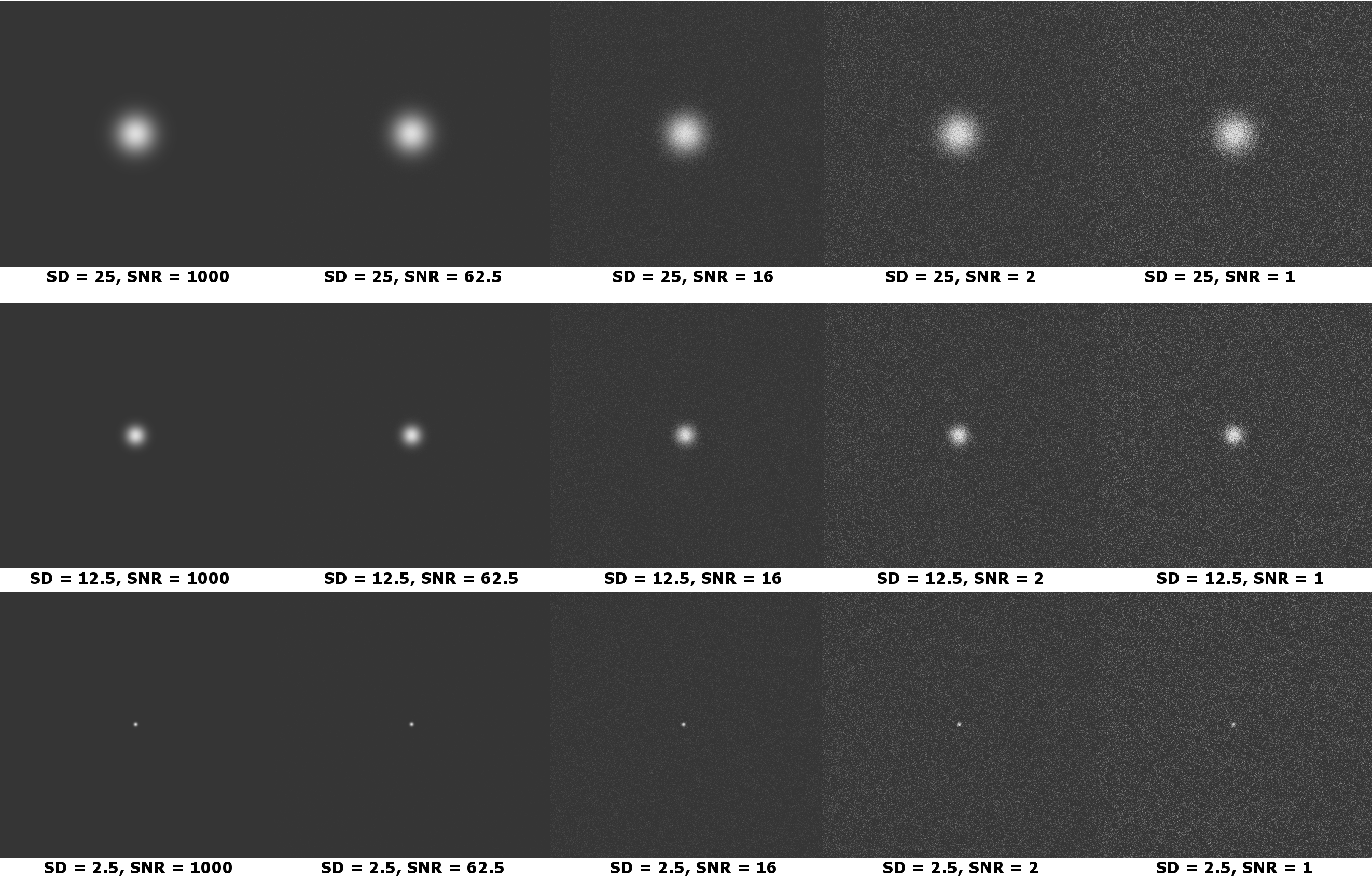

Supplement: S3 Fig — (TIF) [file pone.0175015.s003.tif]

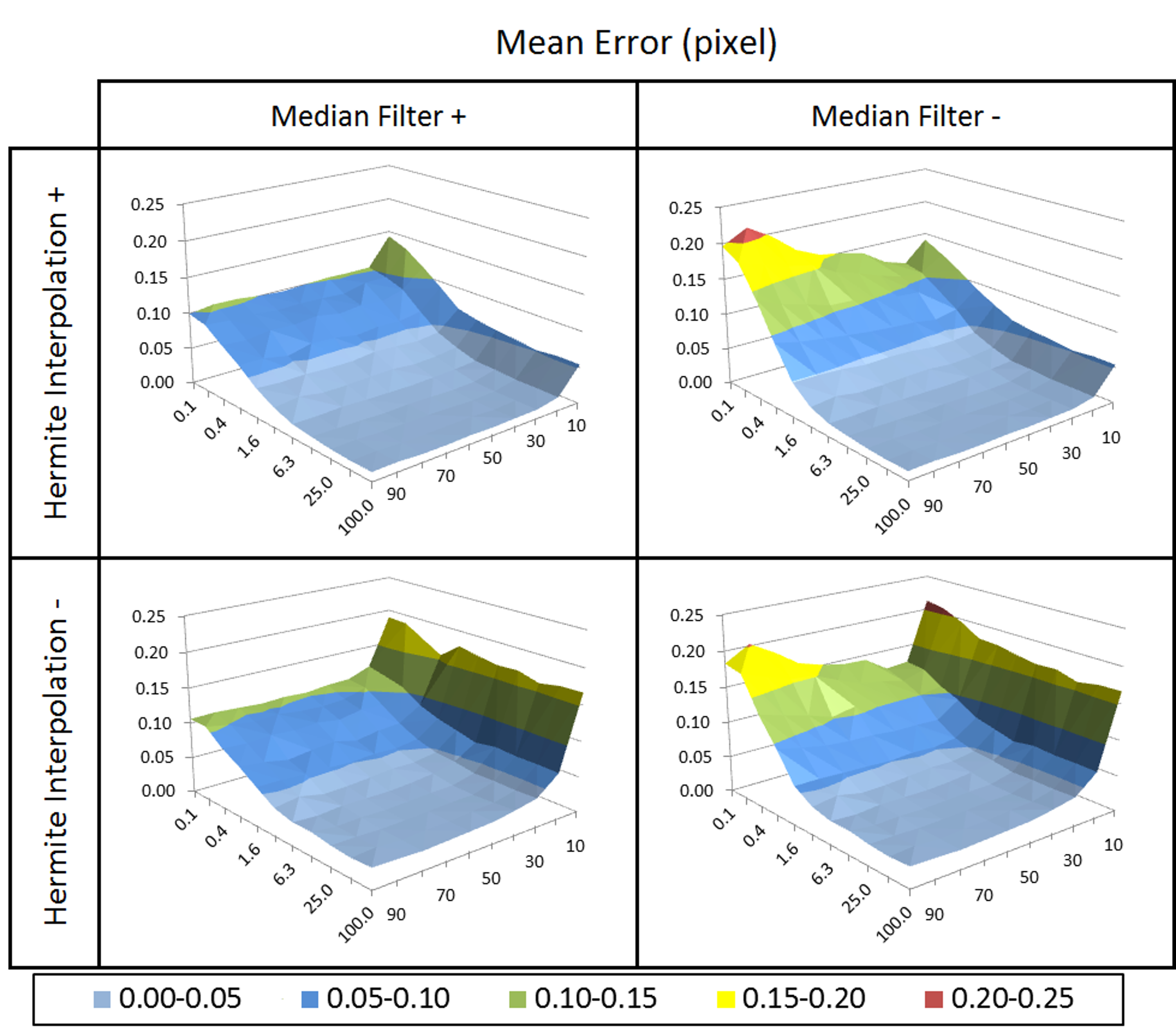

Supplement: S4 Fig — (TIF) [file pone.0175015.s004.tif]

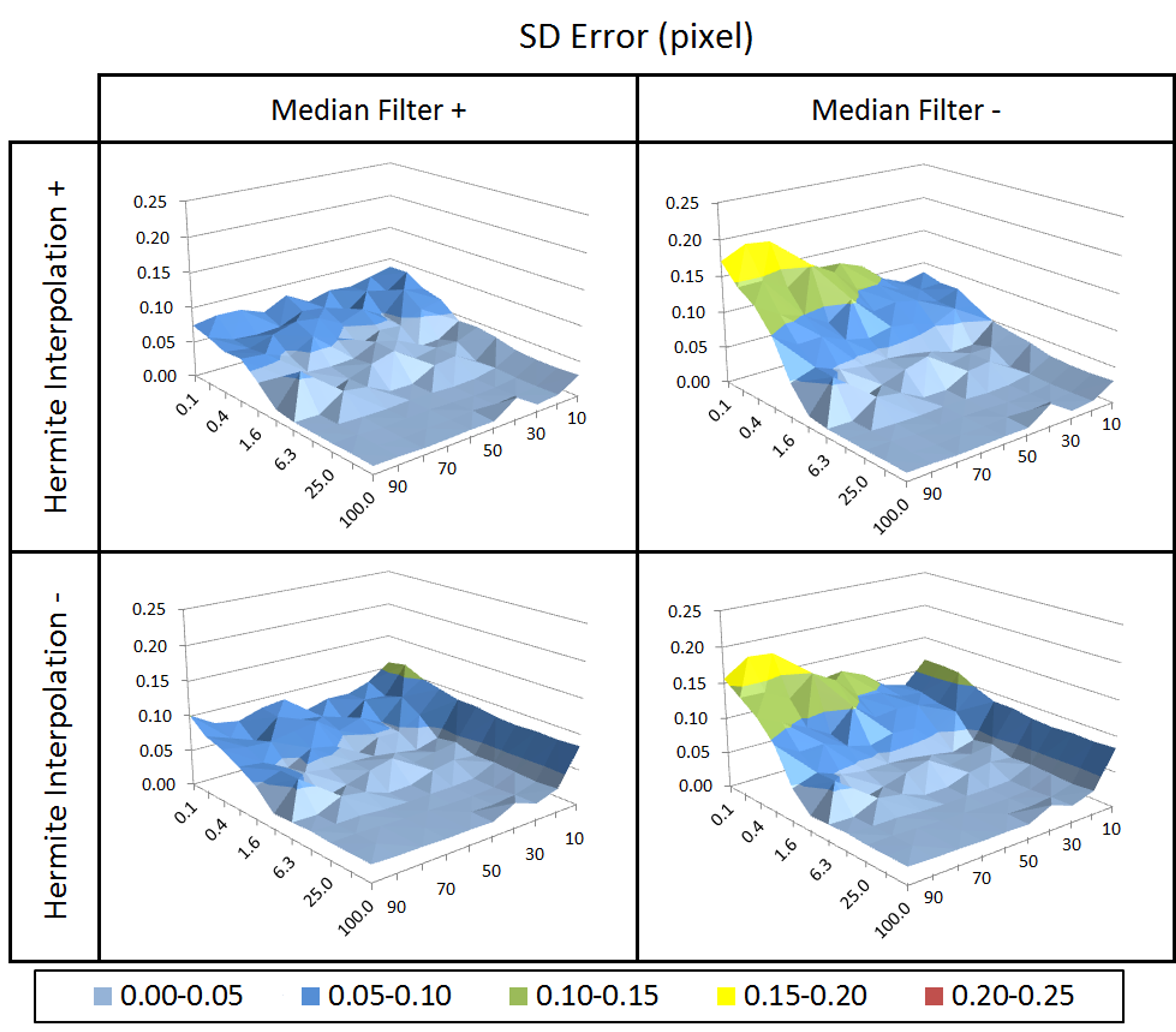

Supplement: S5 Fig — (TIF) [file pone.0175015.s005.tif]

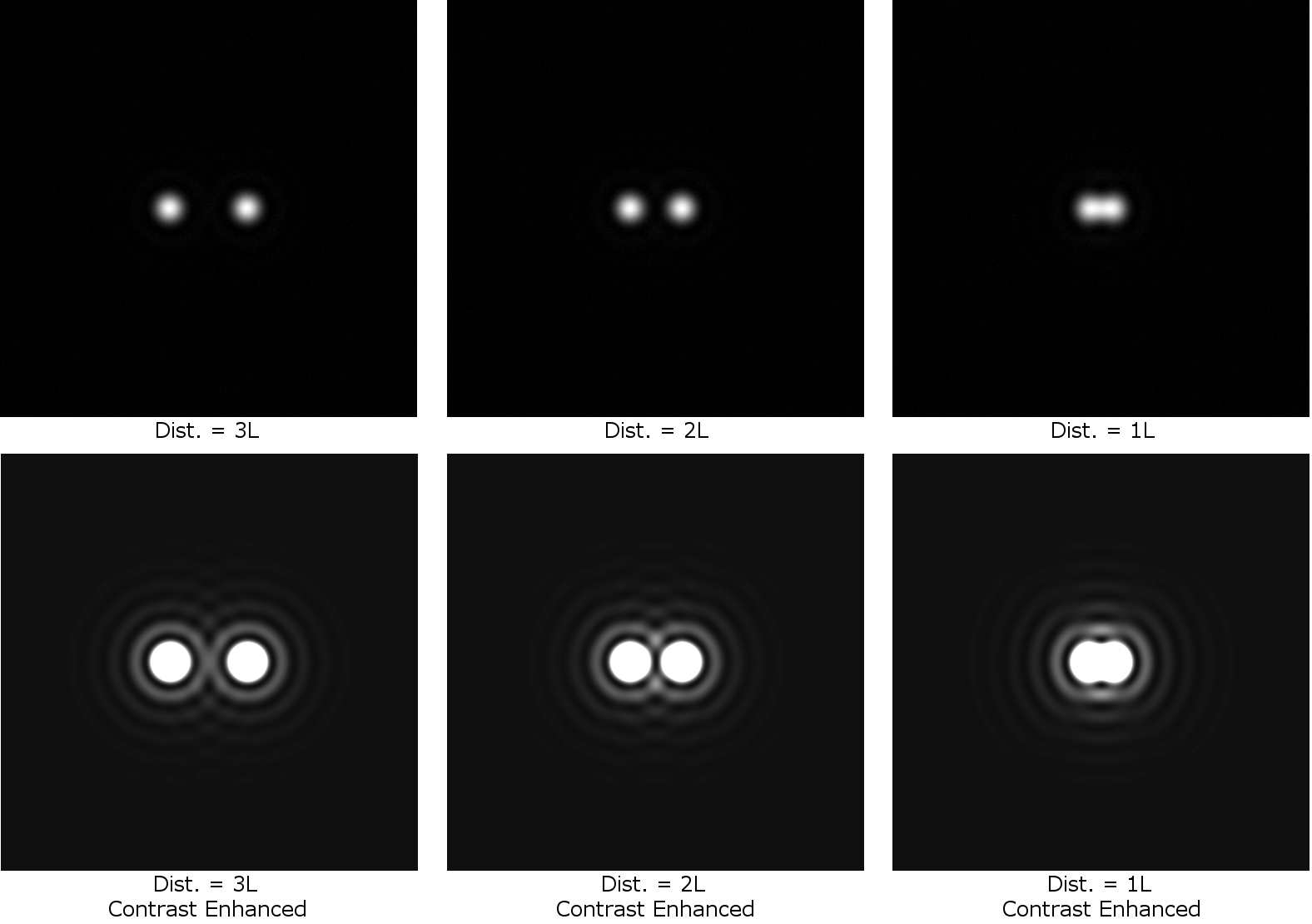

Supplement: S6 Fig — (TIF) [file pone.0175015.s006.tif]

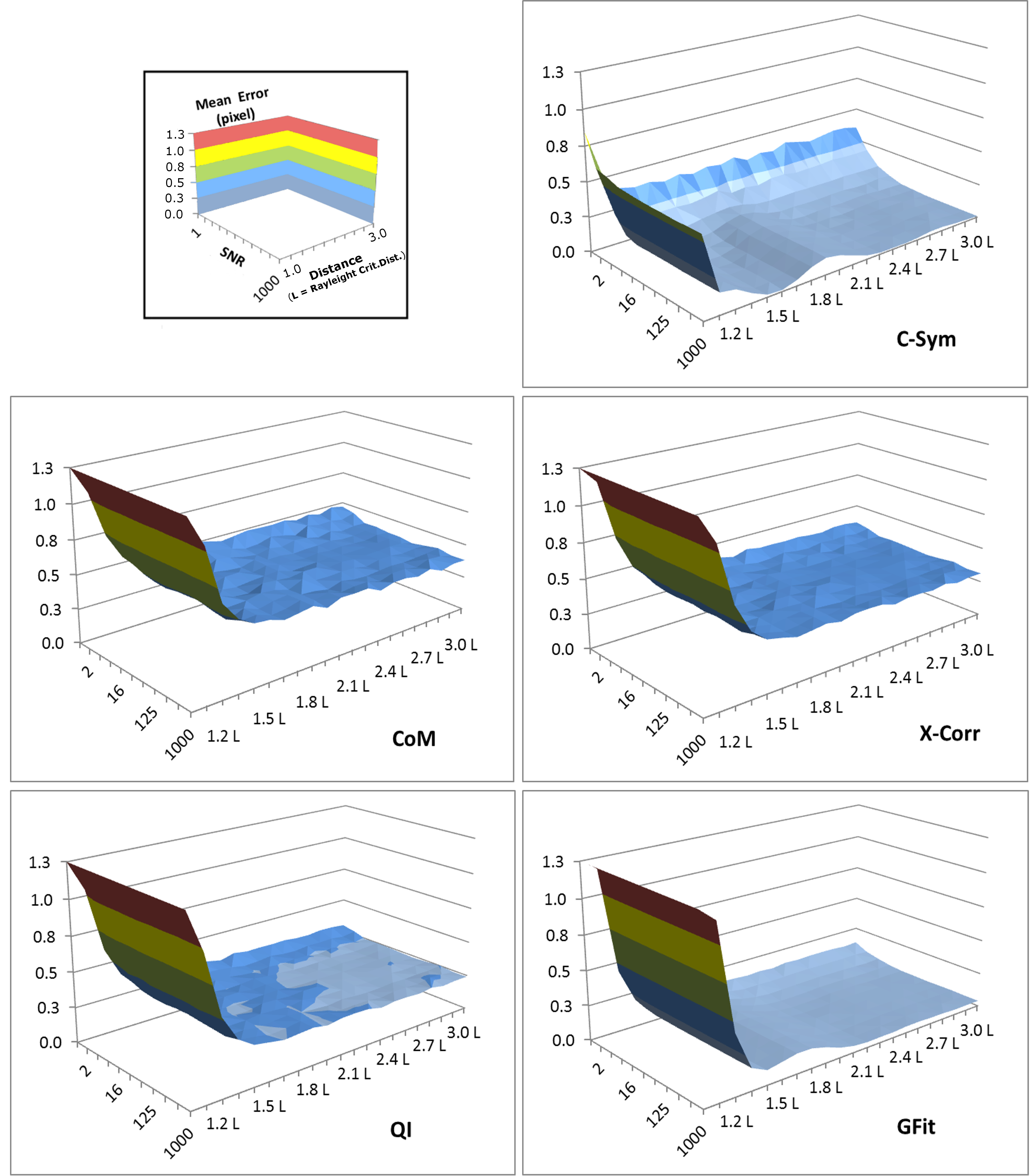

Supplement: S7 Fig — (TIF) [file pone.0175015.s007.tif]

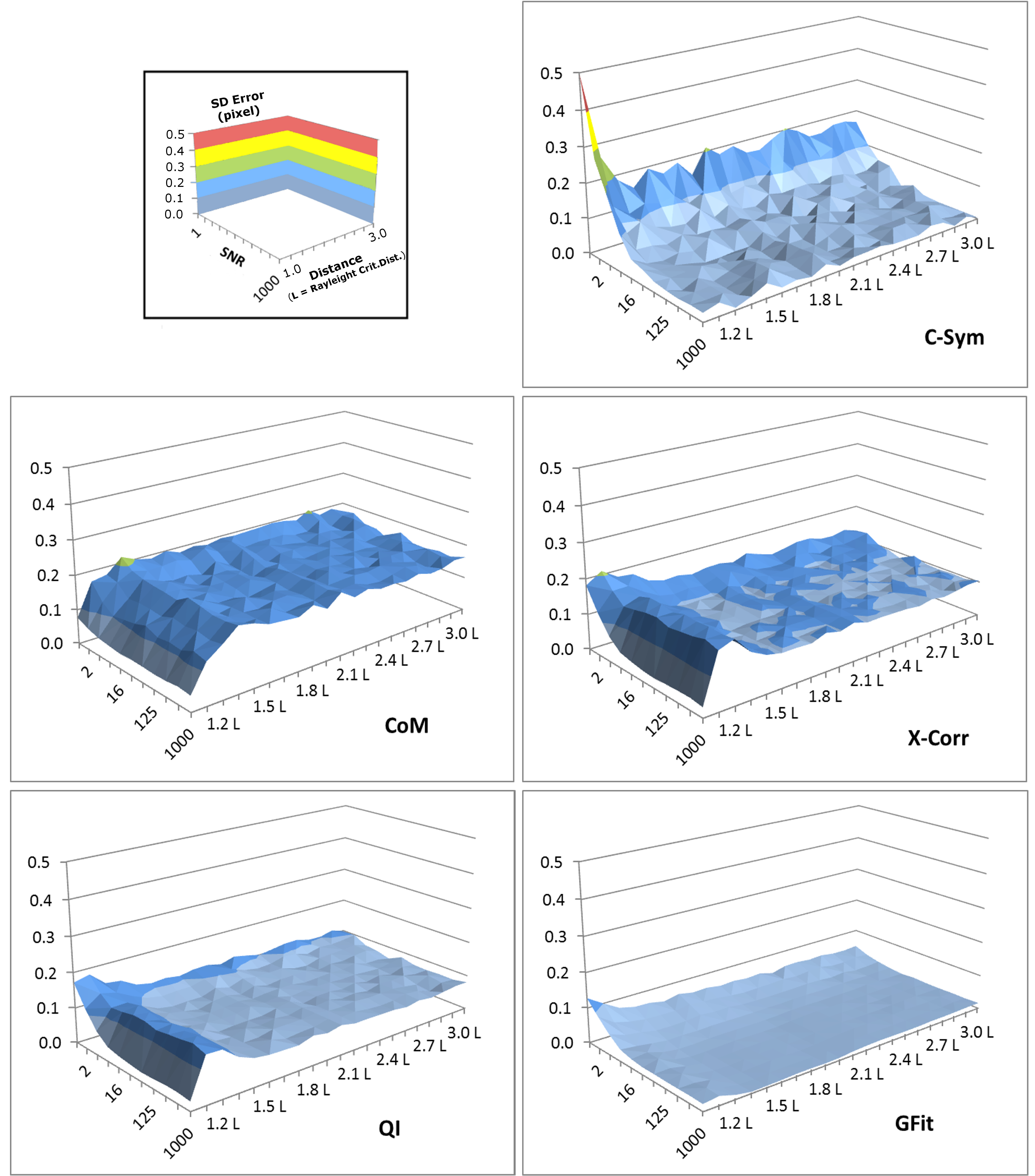

Supplement: S8 Fig — (TIF) [file pone.0175015.s008.tif]
