# Supplementary material for: Refining particle positions using circular symmetry
Source: PLoS One. 2017 Apr 12;12(4):e0175015. doi: 10.1371/journal.pone.0175015 (PMC5389671; doi:10.1371/journal.pone.0175015)
Supplement: S1 File — It contains additional details of the experiment design, the algorithms of this paper, and the additional conducted experiments. (DOCX) [file pone.0175015.s009.docx]

## 1: Design of the experiment with synthetic images

### Synthetic transmitted light microscopy images

To evaluate the accuracy of particle detection we used synthetic images that varied from simple “spots” to complex diffraction patterns. These particles were generated using a diffraction profile extracted from a real particle and by introducing random variations such as; inversion of color, profile scaling and stretching. Also, the background intensity of each image was randomly selected. In this way, we generated for the experiments particles with random appearances and backgrounds and with a selected particle size. Examples of a particle with different SNR levels are presented in S1 Fig. Examples of generated synthetic images with different particle sizes and patterns are presented in the S2 Fig.

### Synthetic fluorescent images

Examples of particles under fluorescent microscopy simulated by a Gaussian distribution function with three different values of standard deviation at several SNR levels are presented in S3 Fig.

## 2: Image processing techniques

Here we briefly describe the fundamentals of the algorithms compared to *C-Sym*.

### Center of Mass (*CoM*)

The center-of-mass is a simple and computational efficient algorithm [1]. The center of mass position (*c_x_, c_y_*), in the X-axis and Y-axis of an image *I*, are calculated as:

| $ROI'\left(i,j\right)=\left\vert ROI\left(i,j\right)-I_{med}\right\vert,$ | () |
| --- | --- |
|  | () |
|  | () |

where (*i, j*) are the pixel coordinates of a *n×n* sized ROI, centered at the initial estimation of the particle position. Since this technique is very sensitive to a non-zero background, the first step is to subtract the median *I*_med_ of the full image from the ROI.

### Gaussian Fitting (*GFit*)

This method is based on approximating the point spread function (the spatial intensity distribution) of a particle with a 2D Gaussian function. In this work, we apply a least-squares Gaussian fitting minimization, expressed as follows

| $\min\sum_{i,j} \left( g\left(i,j\right)-ROI\left(i,j\right)\right)^{2}$, | (4) |
| --- | --- |
| $g\left(i,j\right)=\text{a} e^{-\left(\frac{\left(i-c_{x}\right)}{2\sigma_{x}^{2}}+\frac{\left(j-c_{y}\right)}{2\sigma_{y}^{2}}\right)}$, | (5) |

where (*i, j*) are the pixel coordinates of a *n×n* sized ROI, centered at the initial estimation of the particle position, (*c_x_,c_y_*) represent refined center of the particle, $\text{a}$is the amplitude of the function and represents intensity in the center, and $\left(\sigma_{x},\sigma_{y}\right)$ is the standard and represent the size of the particle. To solve the minimization problem, we have used the Levenberg-Marquardt algorithm [2].

### Circular Hough transform (*CHT*)

The *CHT* is a classic algorithm for finding circles in images. We implemented the *CHT* as explained in detail in [3]. The algorithm works using a range of possible particle radius and the functioning of this algorithm can be summarized as follows:

**Step1:** Edge detection: A binary edge map of the image is created using a standard Sobel edge detector with a gradient threshold calculated automatically using the Otsu technique.

**Step2:** Accumulator array computation: A single 2-D accumulator array is used so pixels of high gradient are designated as being candidate pixels allowed to cast ‘votes' in the accumulator array. Edge pixels cast votes for belonging to circles with a range of particle radius. Edge orientation is also used to permit voting in a limited interval along direction of the gradient.

**Step3:** The voting generates peaks in the accumulator array, and these peaks are evaluated by the algorithm as potential circles.

### Cross-correlation (*XCorr*)

*XCorr* is a method is based on cross correlating an averaged intensity profile with its own mirror for the X and Y axes. Based on empirical experiments, a median background subtraction process was added as a first step, similarly to the *CoM* technique. Formulation of *XCorr* can be expressed as follows:

| $ROI'\left(i,j\right)=\left\vert ROI\left(i,j\right)-I_{\text{med}}\right\vert,$ | (6) |
| --- | --- |
| $P_{X}\left(i\right)=\frac{1}{0.2n}\sum_{j=0.4n}^{0.6n} ROI'(i,j),$ | (7) |
| $P_{Y}\left(j\right)=\frac{1}{0.2n}\sum_{i=0.4n}^{0.6n} ROI'(\mathrm{i},\mathrm{j}),$ | (8) |
| $XCorr_{x}=F^{-1}\left(F\left(P_{X}\left(i\right)\right)\times F\left(P_{X}^{*}\left(-i\right)\right)\right),$ | (9) |
| $XCorr_{Y}=F^{-1}\left(F\left(P_{X}\left(j\right)\right)\times F\left(P_{Y}^{*}\left(-j\right)\right)\right),$ | (10) |

where $I_{med}$ is the image median subtracted to the region of interest ROI centered at the initial estimation of the particle; *P_x_* is the averaged profile of the particle, created using a band of *2n* rows, being *n×n* the size of the ROI. $F$and $F^{-1}$represent the Fourier and inverse Fourier transform; and $\left(P^{*}\left(-i\right)\right)$represent the conjugate transpose of profile matrix $P_{x}$. Here, the particle center *c_x_* is calculated finding the peak of the correlation function using a 5 point parabolic fit to obtain subpixel accuracy.

### Quadrant-Interpolation (*QI*)

QI measurement was introduced in [4]. This algorithm uses the circular geometry of the diffraction pattern to suppress bias and improve a previous measurement applied to the particle. In this work, the *XCorr* measurement is used as a first step of this technique. After this process, the functioning of the technique can be summarized as follows:

**Step1:** The position estimated by the *XCorr* technique is used to calculate a radial profile of the intensity of the particle for each quadrant on a circular grid. Here, it is assumed that the real center of the particle is within ~1 pixel of the previously estimated center. These intensity profiles are created using points spaced by δr and δq, in radial and angular dimensions δr < pixel spacing. To this end, a four neighborhood bilinear interpolation technique is used.

**Step 2:** Relative radial profiles are used to improve the estimated center. Thus, for the X*-*axis, an intensity profile $P_{x}$ is created concatenating the sum of top right and bottom right quadrant profiles with the sum of top left and bottom left ones.

| $q_{R}\left(r\right)=q_{TR}\left(r\right)+q_{BR}\left(r\right),$ | (10) |
| --- | --- |
| $q_{L}\left(r\right)=q_{TL}\left(r\right)+q_{BL}\left(r\right),$ | (11) |
| $P_{x}\left(r\right)=q_{L}\left(-r\right)\cap q_{R}\left(r\right),$ | (12) |
| $QI_{x}=F^{-1}\left(F\left(P_{x}\left(r\right)\right)\times F\left(P_{x}^{*}\left(-r\right)\right)\right),$ | (13) |

where, similarly to *XCorr*, here *q_TR_*(*r*), *q_BR_*(*r*), *q_TL_*(*r*), *q_BL_*(*r*) are top
right, bottom right, top left and bottom left quadrant profiles of the image; represents concatenation;$F$and $F^{-1}$represent the Fourier and inverse Fourier transform; and $F\left(P_{x}^{*}\left(-i\right)\right)$represent the Fourier transform of the complex conjugate. The resulting particle center *c_x_* is calculated finding the peak of the *QI* function using a five-point parabolic fit to obtain subpixel accuracy.

## 3: Central-Symmetry algorithm, median filtering and Hermite Interpolation

We evaluated the response of *C-Sym* in further detail analyzing how a median filtering and the Hermite Interpolation step affect the result of the algorithm. Median Filtering (MF) is a procedure based on replacing each pixel value of an image by its local median. MF has been widely applied to reduce the effects of noise in digital images [5]. To evaluate the noise response, we compared the default version of *C-Sym* with a median filtered version. The piecewise Hermite interpolation algorithm, see Section 3, was included in the algorithm to increase the performance of *C-Sym* with moderate noise values. However, this step can be omitted to decrease computation time. To see how the Hermite interpolation affects the obtained results, we also evaluated *C-Sym* by activating and deactivating this step.

The experiment used 55 000 simulated particle images and was designed as described in section 2.1 by generating 500 simulations for each particle size and noise level. Four versions of the algorithm were therefore evaluated according to its accuracy and precision using the average and standard deviation of the Euclidean distance between the estimated position of the particle and the ground truth. The obtained results are shown in S4 Fig and S5 Fig.

The results show that the Hermite interpolation step generally improves the accuracy of *C-Sym*, though this improvement is only significant in small particles. With SNR < 1, a slightly loss of accuracy may be observed and its use is thus not recommended. The use of median filter improves significantly the results with SNR < 2 with no significant change for higher SNR.

## 4: Overlapping Particles

We generated templates containing two identical patterns representing overlapping micro-particles. This is done by simulating two diffraction patterns produced by light passing through two circular apertures forming Airy diffraction patterns in the image plane using two point-spread functions (Bessel function). We changed the distance between the two particles to measure the effect of the overlapping in accuracy with different levels of noise. Therefore, we calculated the Rayleigh’s criteria, the minimum theoretical distance for two objects to be distinguishable in an optical system*, L,* and selected a range of distances from *L* to 3*L* (S6 Fig).

To avoid bias when generating synthetic images, we set the ground truth position as a random floating-point pixel position. For the initial estimation of particle position, we introduced a random error up to 2 pixels to simulate the labeling or segmentation error. Randomized synthetic images (512x512 pixels) for each particle size with the above mentioned variables were generated. In addition, we added Gaussian noise with zero means and variance $\sigma^{2}$ to the images. To compare the different techniques in the same conditions, we used a constant ROI size of 0.6 times *L*.

The results presented in S7 Fig and S8 Fig shows that, within the range SNR > 2 and Distance > 1.2*L*, *C-Sym* achieved a mean error < 0.3 and the SD of error is < 0.1 pixels. In the same range, *CoM* obtained the least good results, with a mean error around 0.5. *XCorr* and *QI* performed better, but they still obtained a mean error in range [0.3, 0.4] pixels. The *GFit* algorithm showed relatively good results, achieving a mean and standard deviation of error < 0.2 pixels. Notably, all methods do not provide low mean errors when particles distances are close Rayleigh limit *L.* However, *C-Sym* algorithm achieved the minimum number of mean error <0.8 pixels.

# Supporting References

1. Brown LG. A survey of image registration techniques. ACM Comput Surv. 1992;24: 325–376. doi:10.1145/146370.146374

2. More JJ. The Levenberg-Marquardt algorithm: Implementation and theory. Lect Notes Math. 1978;630: 105–116. doi:10.1007/BFb0067700

3. Davies ER. Machine Vision, Third Edition: Theory, Algorithms, Practicalities. Pattern Recognition Letters. 2004.

4. Van Loenhout MTJ, Kerssemakers JWJ, De Vlaminck I, Dekker C. Non-Bias-Limited Tracking of Spherical Particles, Enabling Nanometer Resolution at Low Magnification. Biophys J. Biophysical Society; 2012;102: 2362–2371. doi:10.1016/j.bpj.2012.03.073

5. Narendra PM. A Separable Median Filter for Image Noise Smoothing. IEEE Trans Pattern Anal Mach Intell. 1981;PAMI-3: 20–29. doi:10.1109/TPAMI.1981.4767047
